# An Improved Hybrid Encoding Cuckoo Search Algorithm for 0-1 Knapsack Problems

**DOI:** 10.1155/2014/970456

**Published:** 2014-01-12

**Authors:** Yanhong Feng, Ke Jia, Yichao He

**Affiliations:** ^1^School of Information Engineering, Shijiazhuang University of Economics, Shijiazhuang 050031, China; ^2^School of Information Science and Engineering, Hebei University of Science and Technology, Shijiazhuang 050018, China

## Abstract

Cuckoo search (CS) is a new robust swarm intelligence method that is based on the brood parasitism of some cuckoo species. In this paper, an improved hybrid encoding cuckoo search algorithm (ICS) with greedy strategy is put forward for solving 0-1 knapsack problems. First of all, for solving binary optimization problem with ICS, based on the idea of individual hybrid encoding, the cuckoo search over a continuous space is transformed into the synchronous evolution search over discrete space. Subsequently, the concept of confidence interval (CI) is introduced; hence, the new position updating is designed and genetic mutation with a small probability is introduced. The former enables the population to move towards the global best solution rapidly in every generation, and the latter can effectively prevent the ICS from trapping into the local optimum. Furthermore, the greedy transform method is used to repair the infeasible solution and optimize the feasible solution. Experiments with a large number of KP instances show the effectiveness of the proposed algorithm and its ability to achieve good quality solutions.

## 1. Introduction

The combinatorial optimization plays a very important role in operational research, discrete mathematics, and computer science. The knapsack problem is one of the classical combinatorial optimization problems that are difficult to solve and it has been extensively studied since the pioneering work of Dantzig [1]. Generally speaking, if the classification of these methods that are used to solve such problems is based on the nature of the algorithm, they can be simply divided into two categories [2]: exact methods and heuristic methods. Exact methods, like enumeration method [3, 4], branch and bound [5], and dynamic programming [6], can give the exact solutions; nevertheless, in the worst case, it is required to take a long time to get a satisfactory solution; sometimes the time increases exponentially with the increment of the size of the instance.

Recently, nature-inspired metaheuristic algorithms perform powerfully and efficiently in solving the diverse optimization problems, including combinatorial problem. Metaheuristic algorithms include genetic algorithm [7], particle swarm optimization [8], ant colony optimization [9], artificial bee colony algorithm [10], differential evolution algorithm [11], harmony search algorithm [12, 13], and krill herd algorithm [14–16].

As is mentioned above, metaheuristic methods have been proven to be an effective means to cope with the combinatorial optimization problems including 0-1 knapsack problem. Unlike deterministic search approaches which have the drawbacks of being trapped into local minima unavoidably, the main advantage of metaheuristic methods can deliver satisfactory solutions in a reasonable time. Because of this, it is crucial to present some new nature-inspired methods to deal with the 0-1 knapsack problem and especially to tackle some intractable and complex large-scale instances which are closer to practical applications.

Cuckoo search (CS), a population-driven nature-inspired metaheuristic algorithms originally proposed by Yang and Deb in 2009 and 2010 [17, 18], which showed some promising efficiency for global optimization and is becoming a new research hotspot in evolutionary computation. CS is inspired by the brood parasitism of some cuckoo species by laying their eggs in the nests of other host birds. Each egg (nest or cuckoo) represents a solution, and a cuckoo egg represents a new solution. The aim is to use the new and potentially better solutions (cuckoos) to replace a not-so-good solution in the nests [19]. Like other metaheuristic algorithms, CS uses no gradient information during the search so that it has the ability to solve nonconvex, nonlinear, nondifferentiable, and multimodal problems. Furthermore, there is essentially only a single parameter *p*_*a*_ in CS and thus it is potentially more generic to adapt to a wider class of optimization problems [19]. In addition, Yang and Deb showed that the CS outperforms particle swarm optimization or genetic algorithms in some real-world optimization problems [18, 20]. In virtue of its simplicity, robustness, and so on, books and articles on the subject have proliferated recently. The CS has received more and more attention and application and it falls into a large number of areas [20–25]. More details can be found in [26].

As far as we know, the emphasis of much of previous studies on CS was placed on solving the optimization problems over discrete or continuous space and only a few scholars were concerned about binary problems. In 2011, Layeb [25] developed a variant of cuckoo search in combination with quantum-based approach to solve knapsack problems efficiently. Subsequently, Gherboudj et al. [24] utilized purely binary cuckoo search to tackle knapsack problems. In summary, the studies on binary-coded CS have just begun and its performance needs to further improve so as to further expand its field of application.

Given the above consideration, an improved CS algorithm (ICS) based on the CS framework in combination with a novel greedy strategy is brought forward to solve 0-1 knapsack problem. Compared with the original CS, the outstanding characteristics of Lévy flights such as stability, power law asymptotics used in the original CS is still retained in ICS. Meanwhile, the operation which a fraction of worse nests are abandoned with a probability *p*_*a*_ and new solutions are built randomly is eliminated and a novel operator which the search range is adjusted with adaptive step size and the genetic mutation is embedded is introduced. We assess the performance of our proposed algorithm in terms of the quality of solutions, convergence rate, and robustness by testing twenty different scale knapsack instances. The simulation results not only demonstrated that the proposed algorithm is workable and robust but also held the characteristic of the superior approximation capabilities even in high-dimensional space.

The remainder of this paper is structured as follows.  Section 2 describes the mathematical model for the 0-1 knapsack problems. Then the improvement strategies and the original intention of these improvements are given in detail in Section 3, and the greedy transform method is described. Subsequently, Section 4 presents the results of comparative experiments. Finally, some conclusions and comments are made for further research in Section 5.

## 2. Knapsack Problems

Knapsack problem (KP) is a typical optimization problem and it has high theoretical and practical value. Many practical applications can be formulated as a KP, such as cutting stock problems, portfolio optimization, and scheduling problems, cryptography [27]. This problem has been proven to be a NP-hard problem; hence it cannot be solved in a polynomial time unless P = NP [1]. The classical 0-1 knapsack problem can be defined as follows.

Let *U* = {*u*_1_, *u*_2_, …, *u*_*n*_} be a set of *n* items and *w*_*j*_ and *p*_*j*_ represent the weight and profit of item *j*, respectively. Here, *w*_*j*_, *p*_*j*_, and *j* are all positive integers. The problem is to choose a subset of the items to make their total weight not to exceed a given capacity *C*, while the total profit is maximized. Without loss of generality, it may be assumed that the weight of each item is smaller than the capacity *C* so that each item fits into the knapsack. We can use the binary decision variable *x*_*i*_, with *x*_*i*_ = 1 if item *i* is selected, and *x*_*i*_ = 0 otherwise. The problem can be formulated as follows:

(1) max f=∑i=1npixis.t. ∑i=1nwixi≤C.

## 3. The Improved Cuckoo Search Algorithm (ICS)

Although the original CS algorithm possesses some excellent features of simplicity in structure and escaping from local optima easily compared to several traditional optimization approaches, the phenomenon of slow convergence rate and low accuracy still exists. That is to say, the basic algorithm does not adequately exploit the potential of CS algorithm. Therefore, in this paper, in order to improve the convergence rate and precision of CS, we designed a series of appropriate strategies and then a more efficient algorithm (ICS) is proposed.

ICS introduced the following five improving strategies:

1. using adaptive step size to adjust search range,
2. using confidence interval to enhance the local search,
3. using genetic mutation operation with a low probability to prevent the ICS from being trapped into the local optimum,
4. using hybrid encoding to represent each individual in the population,
5. using greedy transform method to repair the infeasible solution and optimize the feasible solution.

More detailed descriptions of these strategies will be given in subsections, respectively.

### 3.1. Hybrid Encoding

The standard CS algorithm operates in continuous space. Consequently, we cannot use it directly for solving optimization in binary space. Additionally, the operation of the original CS algorithm is closed to the set of real number, but it does not have the closure property in the binary set {0, 1}. Since the wide application of binary optimization problems in real-world engineering, the main objective of the ICS algorithm is to deal with the binary optimization problems. One of the most significant features of the ICS is that it adopts the hybrid coding scheme [28] and each cuckoo individual is represented by two-tuples.

**Definition 1 (auxiliary search space)**

An auxiliary search space *S*′, which denotes a subspace of *n* dimensional real space *R* ^*n*^, where, *S*′ ⊂ *R* ^*n*^. An auxiliary search space *S*′ corresponds to a solution space *S* = {0, 1}^*n*^. Additionally, *S* and *S*′ are two parallel search space. Here the search in *S*′ is called active search; meanwhile, the search in *S* is called passive search.

**Definition 2 (hybrid encoding representation)**

Each cuckoo individual in the population is represented by the two tuples 〈**x**_**i**_, **b**_**i**_〉 (*i* = 1, 2,…, *n*), where **x**_**i**_ works in the auxiliary search space and **b**_**i**_ performs in the solution space accordingly and *n* is the dimensionality of solution. Further, Sigmoid function [26] is adopted to transform a real-coded vector **x**_**i**_ = (*x*_1_, *x*_2_, … , *x*_*n*_)^*T*^ ∈ [−3.0, 3.0]^*n*^ to binary vector **b**_**i**_ = (*b*_1_, *b*_2_, …,*b*_*n*_)^*T*^ ∈ {0, 1}^*n*^. The procedure works as follows:

(2) bi={1if  sig(xi)≥0.50else,

sig(*x*) = 1/(1 + *e* ^−*x*^) is sigmoid function.

### 3.2. Greedy Transform Method

Many optimization problems are constrained. Accordingly, constraint handling is crucial for the efficient design of metaheuristics. Constraint handling strategies, which mainly act on the representation of solutions or the objective function, can be classified as reject strategies, penalizing strategies, repairing strategies, decoding strategies, and preserving strategies [29]. Repairing strategy, most of them are greedy heuristics, can be applied for the knapsack problem [29]. However, the traditional greedy strategy has some disadvantages of solving the knapsack problem [30]. Truong invented a new repair operator which depends on both the greedy strategy and random selection [31]. Although this method can correct the infeasible solution, random selection reduced the efficiency because it was not greedy enough to improve the convergence speed and accuracy. In this paper, a novel greedy transform method (GTM) is introduced to solve this problem [32]. It can effectively repair the infeasible solution and optimize the feasible solution.

This GTM consists of two stages. The first stage (called RS) examines each variable in descending order of *p*_*i*_/*w*_*i*_ and confirms the variable value of one as long as feasibility is not violated. The second stage (called OS) changes the remaining variable from zero to one until the feasibility is violated. The purpose of the OS stage is to repair an abnormal chromosome coding to turn into a normal chromosome, while the RS stage is to achieve the best chromosome coding. Then according to the mathematical model in Section 2, pseudo code of the GTM is described in Algorithm 1.

### 3.3. New Position Updating with Adaptive Step and Genetic Mutation

An outstanding characteristic of PSO is that the individual tends to mimic its successful companion. Each individual follows the simple act that is to emulate the successful experience of the adjacent individual, and the accumulation behavior is to search for the best area for a high-dimensional space [33]. Compared with the PSO, there are some differences and similarities. Firstly, for the PSO, the particle velocity consists of three parts: the previous speed entry, cognitive component, and social composition. The role of social composition of the particles is pulled the direction of the global optimum. For the CS algorithm, new cuckoo individual is generated by a probability *p*_*a*_ in a completely random manner, which can be seen as the social component of the CS. However, it does not well reflect the impact of the entire population on the individual. Secondly, PSO demonstrate adaptive behavior, because the population state is changed in pace with the individual optimum and the global optimum which have been traced. However, cuckoo individual does not fully show the adaptive behavior in the CS algorithm. Thirdly, in PSO, position update formula performs mutation in an embedded memory manner, which is similar to that is used in CS. From the above analyses, we can come to a conclusion that the CS algorithm also has some minor disadvantages. Inspired by the idea of particle swarm optimization, a novel position updating operator is proposed and utilized to strengthen the ability of local search. The ICS and the CS are different in two aspects as follows.

1. The position updating with adaptive step in ICS replaces the random walk completely in CS in the stage of local search.
2. The probability *p*_*a*_ of alien eggs found by host birds is excluded from the CS, and genetic mutation probability (*p*_*m*_) is included in the ICS.

The concept of “confidence interval” is introduced firstly, and the schematic is given as well.

**Definition 3 (confidence interval)**

**Figure 1:**
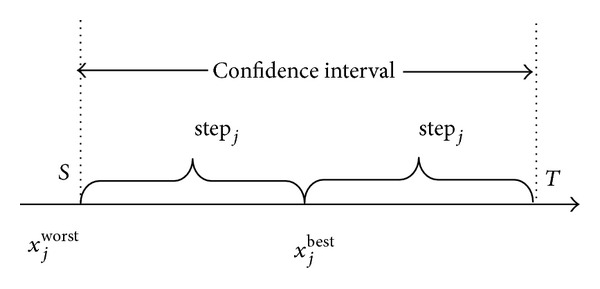
The schematic representation of confidence interval.

Let *x*_*j*_^best^(*t*) be the *j*th component of **x** ^**best**^ ingeneration *t*, and **x** ^**best**^ = (*x*_1_^best^, *x*_2_^best^,…, *x*_*n*_^best^) is the global best cuckoo individual in generation *t*. Let *x*_*j*_^worst^(*t*) be the *j*th component of **x** ^**worst**^ ingeneration *t*, and **x** ^**worst**^ = (*x*_1_^worst^, *x*_2_^worst^, …, *x*_*n*_^worst^) is the global worst cuckoo individual in generation *t* accordingly. The step_*j*_ = |*x*_*j*_^best^ − *x*_*j*_^worst^| is the adaptive step of the *j*th component of individual **x**_**i**_ = (*x*_*i*1_, *x*_*i*2_, …,*x*_*in*_)^*T*^, and then the confidence interval (CI) of every component *x*_*ij*_  (*j* = 1, 2, …, *n*) of **x**_**i**_  (*i* = 1, 2, …, *m*) is defined as CI_*ij*_ ∈ [−step_*j*_, step_*j*_].  Figure 1 gives the schematic representation of confidence interval.

Two major components of any metaheuristic algorithms are intensification and diversification or exploitation and exploration [19], and their interaction can have a marginal effect on the efficiency of a metaheuristic algorithm. The confidence interval is essentially a region near the global best cuckoo. It is significant that the search step size is adjusted gradually in the evolutionary process, which can effectively balance the contradictions between exploration and exploitation. In the early stage of search, cuckoo individuals randomly distributed in the entire response space, so most adaptive steps are large and most confidence intervals are wide, which is very beneficial to making a lot of exploration. As the iterations continue, most adaptive steps gradually become small and most confidence intervals become wide accordingly. Thus the exploitation capabilities will be gradually strengthened.

The purpose of mutation is to introduce new genes so as to increase the diversity of the population. Mutation can also play a balanced exploration-exploitation contradictory role. Genetic mutation operation with a small probability is carried out, for it can effectively prevent the premature convergence of the ICS. New position updating formula of ICS is shown in Algorithm 2.

Here, “best” and “worst” are the indexes of the global best cuckoo and the worst cuckoo, respectively. And *r*, *r*_1_ and rand (·) are all uniformly generated random numbers in [0, 1].

Based on the above-mentioned analyses, the pseudo code of the ICS for 0-1 knapsack problems is described as shown in Algorithm 3.

The time complexity of our proposed algorithm is approximately *O*(max *T*∗*m*∗2*n*) + *O*(*n*log *n*) and it is still linear. The time complexity of new proposed algorithm does not increase in magnitude compared with the original CS algorithm.

## 4. Experimental Results and Analysis

In order to test the optimization ability of ICS and investigate effectiveness of the algorithms for different instance, types, we consider twenty 0-1 knapsack problems involving ten small-scale instances, six medium-scale instances and four large-scale instances. The solution quality and performance are compared with binary version HS and binary version CS, for simplicity, denoted as HS and CS, respectively.

Test problems 1–10 are taken from [12]. Test problems 11 and 13 of He et al. [28] are used in our numerical experiments. Test problem 15 is conducted from test problems 11 and 13. Test problem 16 is generated by Kellerer et al. [34]. Test problems 12 and 14 are generated by Gherboudj et al. [24]. Test problems 17–20 are taken from [12].

All the algorithms were implemented in Visual C++ 6.0. The test environment is set up on personal computer with AMD Athlon(tm) II X2 250 Processor 3.01 GHz, 1.75 G RAM, running on Windows XP. Three groups of experiments were performed to assess the efficiency and performance of our algorithm. In all experiments, we have set the parameters of CS as follows: *p*_*a*_ = 0.25, the number of cuckoo is 20. For the HS algorithm, harmony memory size HMS = 5, harmony memory consideration rate HMCR = 0.9, pitch adjusting rate PAR = 0.3, and bandwidth *b*_*w*_ = *x*_*U*_ − *x*_*L*_. For the ICS algorithm, the number of cuckoo is 20, the generic mutation probability *p*_*m*_ = 0.15. The experiments on each function were repeated 30 times independently. The quantification of the solutions is tabulated in Tables 1–3.

### 4.1. Comparison among Three Algorithms on Small Dimension Knapsack Problems

Table 1 shows the experimental results of our ICS algorithm, the HS, and the CS on ten KP tests with different dimension. Observation of the presented results in Table 1 indicates that the proposed ICS algorithm performs better than HS algorithm and CS algorithm in *f*8. The optimal solution of the test problem 8 found by ICS is x* = (1, 1, 1, 1, 1, 1, 1, 1, 1, 0, 1, 0, 1, 0, 0, 0, 0, 0, 0, 0, 0, 0, 0) and *f*8(x*) = 9777. Additionally, CS and ICS have the same results in *f*6 which is better than that obtained by the HS algorithm and three algorithms have the same results in the other instances. In a word, the solutions obtained by three algorithms are similar and there is almost no significant difference among all the three algorithms. Further, ICS algorithm does not show its advantages thoroughly. Therefore, in order to further test the performance of the algorithm, we conducted the following experiments in the next subsection.

### 4.2. Comparison among Three Algorithms on Medium Dimension Knapsack Problems

**Figure 2:**
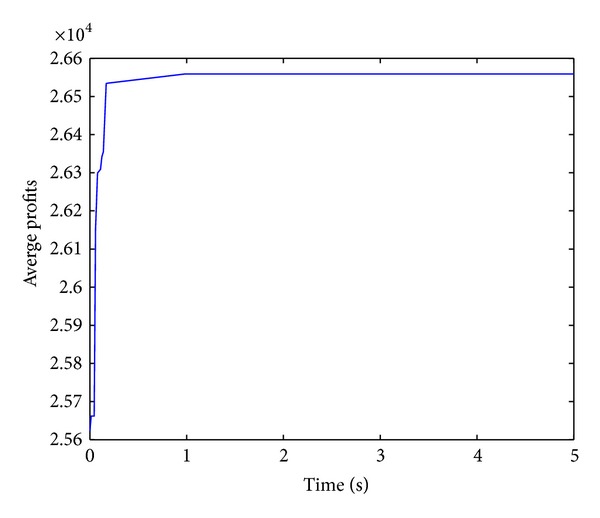
Best profits (100 items).

**Figure 3:**
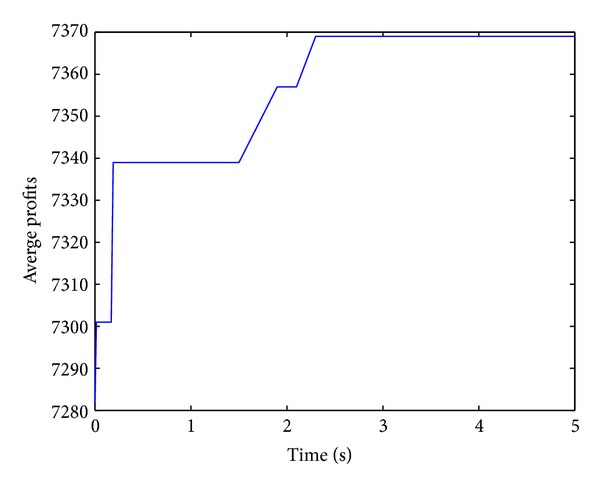
Best profits (120 items).

**Figure 4:**
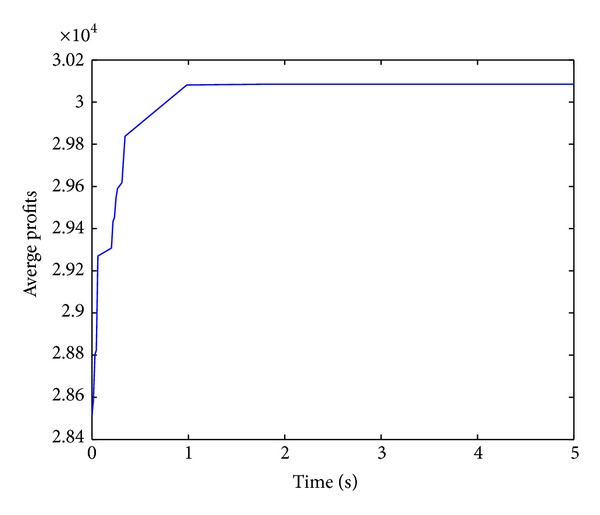
Best profits (150 items).

**Figure 5:**
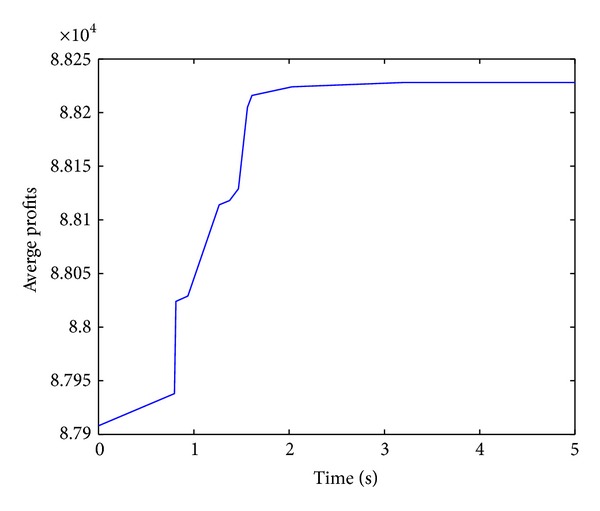
Best profits (200 items).

Figures 2, 3, 4, and 5 show convergence curves of the best profits of the ICS over 30 runs on four test problems with 100, 120, 150, and 200 items. It indicates the global search ability and the convergence ability of the ICS. There are several observations and they are given as follows.

The best profit of 100 items test problem is quickly increasing and reaching the approximately optimal profit at nearly one second. Although the algorithm shows a slow evolution only for a moment in 120 items, the best profit is still obtained after about 2.5 seconds. In the 150 items test problem, the best profit is quickly increasing for more than a second. For the large 200 items test problem, the best profit is also increasing very rapidly over 2.5 seconds. The performance of the ICS can be further understood and analyzed from Table 2.

We observed from Table 2 that the ICS has demonstrated an overwhelming advantage over the other two algorithms on solving 0-1 knapsack problems with medium scales. ICS and HS obtained the same optimal solution in all test problems. The CS has the worst performance, and the best solutions found by CS are worse than those obtained by the other two algorithms for *f*12 and *f*15. Furthermore, the worst solutions found by the ICS are all better than those obtained by CS. The ICS and the HS obtained the same worst solutions except *f*13 and *f*16. Unfortunately, the worst solution obtained by ICS cannot exceed that of the HS. The ICS uses little “time” and little “average time” compared with CS for almost all of the test problems. In addition, the “*G*_best_/*G*_avg_” of most problems is much smaller than that of the CS and the HS, which shows that the ICS has a fast convergence. “SR” is more than 95% for almost all of problems except *f*15 and *f*16. Further, “SR” for *f*15 and *f*16 is slightly higher than that of other two algorithms, which indicates the high efficiency of the ICS on solving 0-1 knapsack problems. “Std.dev” is much smaller than that of the CS and the difference is not very poor between ICS and HS, which indicates the good stability of the ICS and superior approximation ability.

**Figure 6:**
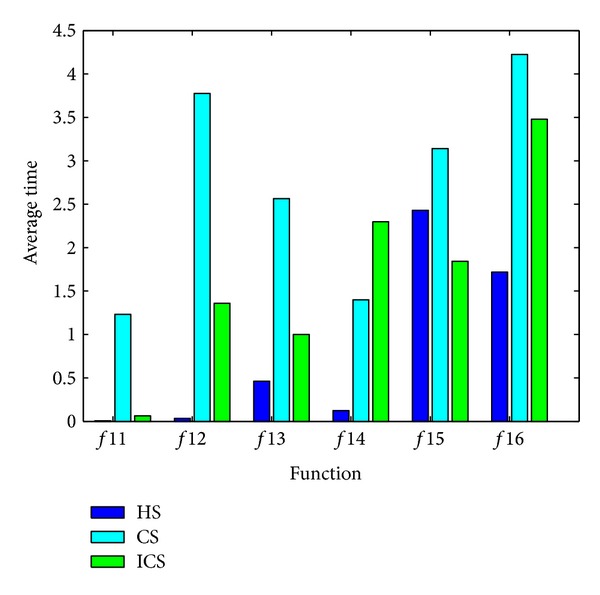
Comparison of average computation time of the ICS with the HS and the CS.

Figure 6 shows a comparison of average computation time with *f*11 to *f*16, estimated by seconds for the proposed algorithms, the HS and the CS. In terms of the average computation time, Figure 6 shows that the HS algorithm is the best one and the CS algorithm is the worst one. Moreover, ICS converges to the optima faster than CS on most instances.

Although ICS has shown some advantages on solving 0-1 knapsack problem with medium-scale instances; however, the optimal solution obtained by ICS is not very prominent compared with other two algorithms. Therefore, in order to further verify the efficiency of our proposed algorithm, we designed the large scale knapsack tests as follows.

### 4.3. Comparison among Three Algorithms on Solving 0-1 Knapsack Problems with Large Dimension

Similar to the results of medium-scale knapsack instances, for the large scale knapsack problem, we observe that the ICS algorithm obtains better solutions in shorter time and has more obvious advantages over the CS algorithm from Table 3. Regrettably, ICS is slightly inferior to HS in terms of the optimal solution quality on function 17 and function 20. In a word, the ICS has demonstrated better performance and it thus provides an efficient alternative on solving 0-1 knapsack problems.

**Figure 7:**
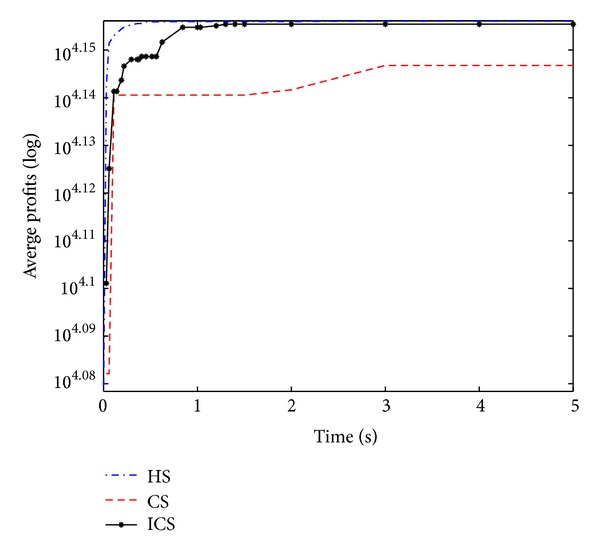
The performance on function 17.

**Figure 8:**
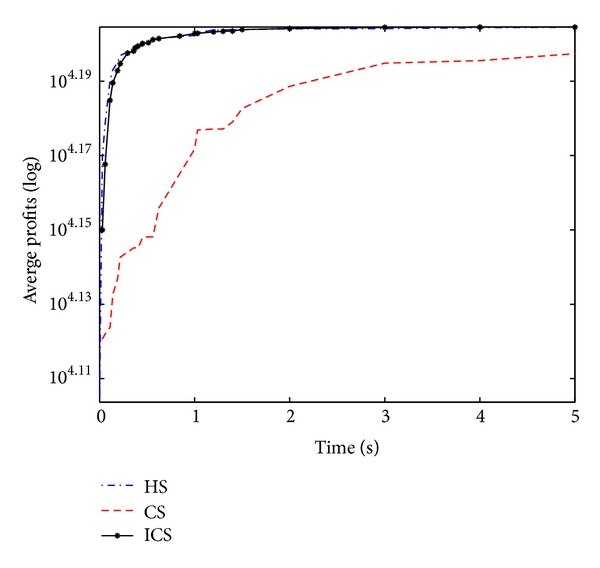
The performance on function 18.

**Figure 9:**
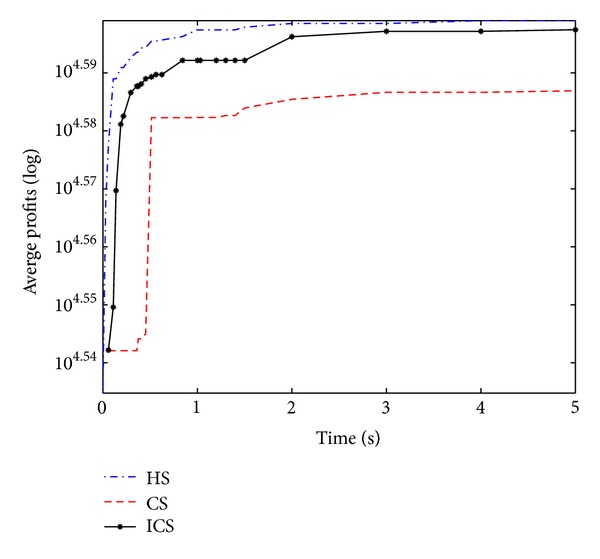
The performance on function 19.

**Figure 10:**
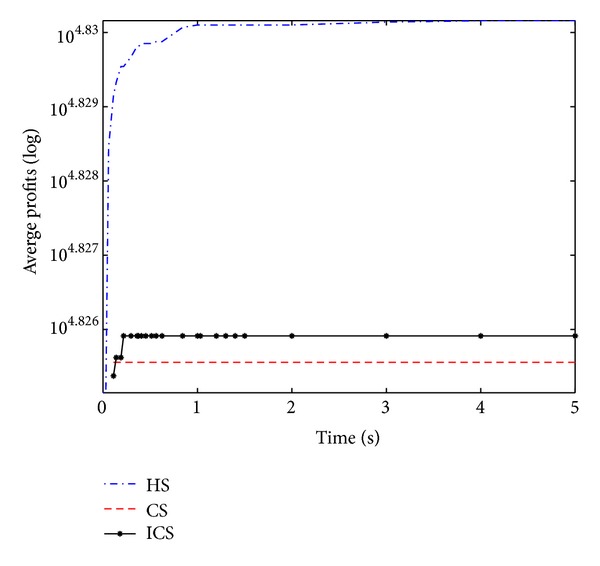
The performance on function 20.

Convergence curves shown in Figures 7, 8, 9, and 10 similarly establish the fact that ICS is more effective than CS in all four large-scale KP instances. Through careful observation, it can be seen that HS gets outstanding profits in the initial stage of the evolution and the best value in the final population. Compared with HS and ICS, CS obtained the worst mean profits at various stages. ICS and HS have roughly the same convergence speed. In addition, it is obvious to infer that CS and ICS get stuck at local optima quickly as can be seen from Figure 10. However, HS converges to the global optimum rapidly.

## 5. Conclusions

In this paper, the ICS algorithm has been proposed based on the CS framework and a greedy transition method to solve 0-1 knapsack problem efficiently. An adaptive step is carefully designed to balance the local search and the global search. Genetic mutation operator helps the algorithm to yield fast convergence and avoids local optima. The simulation results demonstrate that the proposed algorithm has superior performance when compared with HS and CS. The proposed algorithm thus provides a new method for solving 0-1 knapsack problems.

Further studies will focus on the two issues. On one hand, we would apply our proposed approach on other combinatorial optimization problems, such as multidimensional knapsack problem (MKP) and traveling salesman problem (TSP). On the other hand, we would examine new meta-hybrid to solve 0-1 knapsack problems which are too complicated.

## Figures and Tables

**Algorithm 1:**
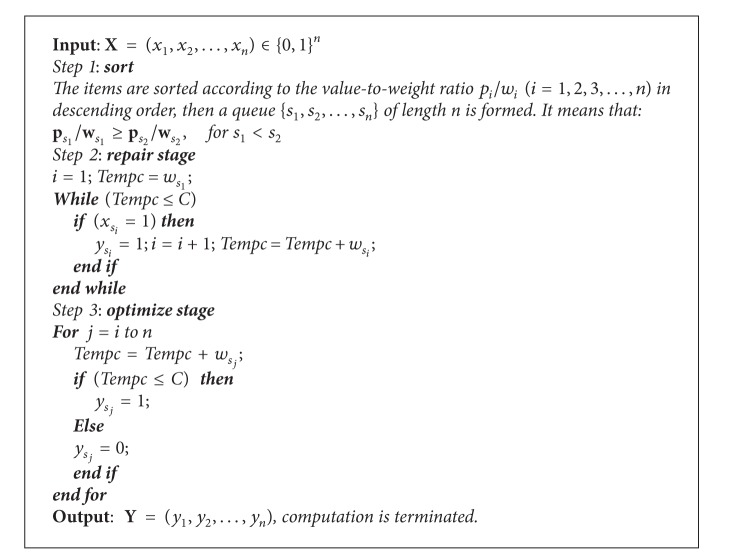
Greedy transform method.

**Algorithm 2:**
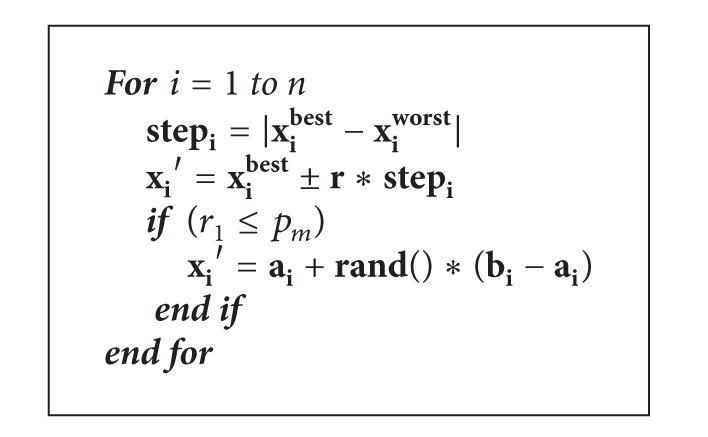
New position updating formula of ICS.

**Algorithm 3:**
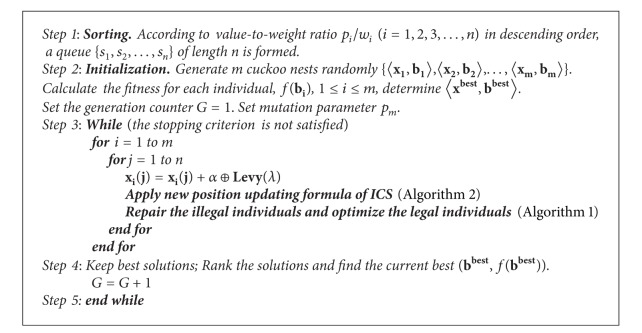
The main procedure of ICS.

**Table 1:** Experimental result of three algorithms with small KP instances.

Fun	Size	HS	CS	ICS
*f*1	10	295	295	295
*f*2	20	1024	1024	1024
*f*3	4	35	35	35
*f*4	4	23	23	23
*f*5	15	481.0694	481.0694	481.0694
*f*6	10	50	**52**	**52**
*f*7	7	107	107	107
*f*8	23	9761	9776	**9777**
*f*9	5	130	130	130
*f*10	20	1025	1025	1025

**Table 2:** Experimental result of three algorithms with medium KP instances.

Fun	Dim	Algorithm	*V*_best_/*C*_best_	*V*_worst_/*C*_worst_	*T*_best_/*T*_avg_	*G*_best_/*G*_avg_	Mean	Std. dev	SR
*f*11	50	HS	3103/1000	3103/1000	0.00/0.007	96/498	3103	0	100
		CS	3103/1000	3097/1000	0.015/1.231	6/502	3102	20.09	95
		ICS	3103/1000	3103/1000	0.00/0.064	2/22	3103	0	100

*f*12	80	HS	9201/1505	9199/1505	0.00/0.033	188/1445	9200	0.45	95
		CS	9199/1505	9106/1505	2.924/3.776	757/980	9176	26.27	15
		ICS	9201/1505	9199/1505	0.015/1.359	5/315	9200	1.03	50

*f*13	100	HS	26559/6717	26559/6717	0.00/0.460	150/16521	26559	0	100
		CS	26559/6717	25882/6713	1.719/2.565	351/529	26447	178.58	20
		ICS	26559/6717	26534/6706	0.062/0.999	12/182	26558	5.59	95

*f*14	120	HS	7393/1109	7393/1109	0.00/0.123	52/3787	7393	0	100
		CS	7393/1109	7334/1109	1.328/1.399	151/159	7361	13.88	6
		ICS	7393/1109	7393/1109	0.344/2.298	53/355	7393	0	100

*f*15	150	HS	30085/7718	30081/7718	0.578/2.429	16140/68532	30081	1.23	10
		CS	30081/7718	29943/7717	0.656/3.142	182/872	30072	32.13	90
		ICS	30085/7718	30081/7718	1.203/1.843	144/215	30082	1.642	20

*f*16	200	HS	88228/71893	88129/71893	0.921/1.718	19115/35429	88150	41.47	25
		CS	88228/71893	88018/71881	4.200/4.225	868/872	88160	67.42	10
		ICS	88228/71893	88221/71886	1.570/3.479	145/323	88225	2.97	30

**Table 3:** Experimental results of three algorithms with large KP instances.

Fun	Dim	Algorithm	*V*_best_/*C*_best_	*V*_worst_/*C*_worst_	*T*_best_/*G*_best_	Mean	Std. dev
*f*17	300	HS	14328/1700	14307/1700	3.391/50277	14325	5.23
		CS	14306/1696	13027/1698	3.422/239	14021	275.06
		ICS	14318/1700	14279/1698	2.125/131	14303	10.61

*f*18	500	HS	16031/2000	15989/1999	1.828/17885	16015	11.21
		CS	16009/2000	15353/2000	2.500/104	15755	174.70
		ICS	16042/2000	15991/1999	2.156/79	16016	13.76

*f*19	800	HS	39759/5000	39656/5000	3.812/22659	39720	28.75
		CS	38987/4999	38296/4998	3.218/83	38630	191.60
		ICS	39775/4995	39432/4996	3.968/91	39578	85.06

*f*20	1000	HS	67635/10000	67630/10000	3.11/15208	67633	1.59
		CS	66992/9997	66712/9998	0.109/1	66877	70.68
		ICS	67123/10000	66975/10000	0.687/13	67042	41.25

## References

[1] Dantzig GB (1957). Discrete-variable extremum problems. *Operations Research*.

[2] Jourdan L, Basseur M, Talbi E-G (2009). Hybridizing exact methods and metaheuristics: a taxonomy. *European Journal of Operational Research*.

[3] Cabot AV (1970). An enumeration algorithm for knapsack problems. *Operations Research*.

[4] James RJW, Nakagawa Y (2005). Enumeration methods for repeatedly solving Multidimensional Knapsack sub-problems. *IEICE Transactions on Information and Systems*.

[5] Kolesar PJ (1967). A branch and bound algorithm for the knapsack problem. *Management Science*.

[6] Martello S (1990). *Knapsack Problem: Algorithms and Computer Implementations*.

[7] Lin F-T (2008). Solving the knapsack problem with imprecise weight coefficients using genetic algorithms. *European Journal of Operational Research*.

[8] Bansal JC, Deep K (2012). A modified binary particle swarm optimization for knapsack problems. *Applied Mathematics and Computation*.

[9] Kong M, Tian P, Kao Y (2008). A new ant colony optimization algorithm for the multidimensional Knapsack problem. *Computers and Operations Research*.

[10] Kashan MH, Nahavandi N, Kashan AH (2012). DisABC: a new artificial bee colony algorithm for binary optimization. *Applied Soft Computing Journal*.

[11] Wang L, Fu X, Mao Y (2012). A novel modified binary differential evolution algorithm and its applications. *Neurocomputing*.

[12] Zou D, Gao L, Li S, Wu J (2011). Solving 0-1 knapsack problem by a novel global harmony search algorithm. *Applied Soft Computing Journal*.

[13] Guo LH, Wang GG, Wang H (2013). An effective hybrid firefly algorithm with harmony search for global numerical optimization. *The Scientific World Journal*.

[14] Wang GG, Gandomi AH, Alavi AH (2013). An effective krill herd algorithm with migration operator in biogeography-based optimization. *Applied Mathematical Modelling*.

[15] Wang GG, Gandomi AH, Alavi AH (2013). Stud krill herd algorithm. *Neurocomputing*.

[16] Wang GG, Guo LH, Wang H (2012). Incorporating mutation scheme into krill herd algorithm for global numerical optimization. *Neural Computing and Applications*.

[17] Yang X-S, Deb S Cuckoo search via Lévy flights.

[18] Yang X-S, Deb S (2010). Engineering optimisation by cuckoo search. *International Journal of Mathematical Modelling and Numerical Optimisation*.

[19] Yang XS (2010). *Nature-Inspired Metaheuristic Algorithms*.

[20] Chaowanawate K, Heednacram A Implementation of cuckoo search in RBF neural network for flood forecasting.

[21] Chifu VR, Pop CB, Salomie I, Suia DS, Niculici AN (2011). Optimizing the semantic web service composition process using Cuckoo Search. * Intelligent Distributed Computing V*.

[22] Choudhary K, Purohit GN (2011). A new testing approach using cuckoo search to achieve multi-objective genetic algorithm. *Journal of Computing*.

[23] Dhivya M, Sundarambal M, Anand LN (2011). Energy efficient computation of data fusion in wireless sensor networks using cuckoo based particle approach (CBPA). *International Journal of Computer Networks and Security*.

[24] Gherboudj A, Layeb A, Chikhi S (2012). Solving 0-1 knapsack problems by a discrete binary version of cuckoo search algorithm. *International Journal of Bio-Inspired Computation*.

[25] Layeb A (2011). A novel quantum inspired cuckoo search for knapsack problems. *International Journal of Bio-Inspired Computation*.

[26] Yang XS, Deb S (2013). Cuckoo search: recent advances and applications. *Neural Computing and Applications*.

[27] Truong TK, Li K, Xu YM (2013). Chemical reaction optimization with greedy strategy for the 0-1 knapsack problem. *Applied Soft Computing*.

[28] He YC, Wang XZ, Kou YZ (2007). A binary differential evolution algorithm with hybrid encoding. *Journal of Computer Research and Development*.

[29] Talbi EG (2009). *Metaheuristics: From Design To Implementation*.

[30] Kennedy J, Eberhart RC A discrete binary version of the particle swarm algorithm.

[31] Zhao J, Huang T, Pang F, Liu Y Genetic algorithm based on greedy strategy in the 0-1 knapsack problem.

[32] He YC, Liu KQ, Zhang CJ (2007). Greedy genetic algorithm for solving knapsack problems and its applications. *Computer Engineering and Design*.

[33] Engelbrecht AP (2009). *Fundamentals of Computational Swarm Intelligence*.

[34] Kellerer H, Pferschy U, Pisinger D (2004). *Knapsack Problems*.

